# Utilizing ultrasound findings of a single indicator joint to assess non-systemic juvenile idiopathic arthritis

**DOI:** 10.1186/s12969-021-00550-0

**Published:** 2021-04-29

**Authors:** Yung-Hsien Huang, Ya-Chiao Hu, Chun-Hua Liao, Bor-Luen Chiang, Cheng-Hsun Lu, Ko-Jen Li, Yao-Hsu Yang

**Affiliations:** 1 Department of Pediatrics, Fu Jen Catholic University Hospital, Fu Jen Catholic University, New Taipei City, Taiwan; 2 Department of Pediatrics, New Taipei City Hospital, New Taipei City, Taiwan; 3 Department of Pediatrics, National Taiwan University Hospital, College of Medicine, National Taiwan University, Taipei, Taiwan; 4 Department of Internal Medicine, National Taiwan University Hospital, Taipei, Taiwan; 5 Department of Pediatrics, National Taiwan University Hospital, Hsin-Chu Branch, Hsinchu, Taiwan

**Keywords:** Juvenile idiopathic arthritis, Musculoskeletal ultrasound, Indicator joint, Disease activity

## Abstract

**Background:**

Musculoskeletal ultrasound (MSUS) has been used worldwide in adult patients with rheumatoid arthritis (RA) but is beginning to play an increasing role in patients with juvenile idiopathic arthritis (JIA). The aim of this study was to investigate the application of MSUS findings of a single indicator joint in JIA to assess the disease activity and classify disease subtype.

**Methods:**

Thirty-five non-systemic JIA patients with a total of 62 visits were retrospectively recruited in this study. Among the involved joints, the joint with highest value of grey-scale (GS) plus power Doppler (PD) (=GSPD) was selected as the indicator joint at each visit. The correlations between each MSUS parameter (GS, PD, GSPD) of indicator joints and the Physician Global Assessment (PGA) score, the Childhood Health Assessment Questionnaire-disability index (CHAQ-DI), and laboratory data were analyzed. The ultrasound features in different subtypes of JIA were also compared.

**Results:**

PD was weakly correlated with the PGA score (rho = 0.323, *p* = 0.010), while both GS and GSPD were moderately correlated with the PGA score (rho = 0.405, *p* = 0.001; rho = 0.434, *p* = 0.000). On the other hand, GS, PD, and GSPD were weakly correlated with CHAQ-DI. Although erythrocyte sedimentation rate (ESR) and C-reactive protein (CRP) had a weak correlation with PGA, they were not statistically correlated with GS, PD, or GSPD. The proportions of effusion, synovial hypertrophy, and enthesopathy in three different subtypes, showed significant differences (Fisher’s exact test, *p* = 0.037; *p* = 0.004; *p* = 0.019). Enthesopathy was only seen in joints of enthesitis-related arthritis (ERA), but not in joints of polyarthritis and oligoarthritis.

**Conclusions:**

MSUS is an acceptable non-invasive tool for the patients with JIA, particularly for those with non-systemic JIA, that might assist disease classification, and whose parameters of the indicator joints may potentially contribute to the evaluation of disease activity.

**Supplementary Information:**

The online version contains supplementary material available at 10.1186/s12969-021-00550-0.

## Background

Juvenile idiopathic arthritis (JIA) is a chronic inflammatory arthritis that causes arthralgia and decreased ability to function in daily life in pediatric patients [1]. The diagnosis can be difficult and delayed in children who may not clearly express the complaints. Because the joint pain could cause variable problems, including abnormal gaits, refusing to use the affected joint, or a posture of guarding the joints [2, 3], close observation is always necessary. The severity of pain sometimes is not easily evaluated, which could be influenced by many factors, including sex, age, pain threshold, family pain culture, and coping strategies [4]. Together, these make it difficult for parents to objectively evaluate and report the disease severity [5]. Instead of severity evaluation by patients with JIA and/or their caregivers, a questionnaire is designed for them as The Childhood Health Assessment Questionnaire (CHAQ) to evaluate physical functions of patients with JIA.

The Physician Global Assessment (PGA) has been widely used to evaluate disease activity [6], and it is simple for physicians to perform. However, the result could be influenced by the reaction and reporting of pain, discomfort and physical symptoms according to the child’s experience of medical personnel [7]. In our clinical experience, the PGA was sometimes difficult to be successfully conducted in those patients who could not cooperate well with the physicians during physical examinations, especially in the young children and toddlers. Therefore, it would be better if an objective, quick and non-invasive tool that could be applied in disease activity assessment for JIA patients.

Musculoskeletal ultrasound (MSUS) has been widely employed in adult patients with rheumatoid arthritis (RA). MSUS could help physicians to make diagnosis of synovitis in RA. MSUS findings have good correlations with classical measures of clinical activity [8]. In JIA, the utility of MSUS is just emphasized gradually in recent years. It has been shown to be more sensitive than other clinical examinations in detecting synovitis and enthesitis [9–13]. There is a growing number of evidences suggesting the correlations between clinical features and MSUS findings [14–17].

In this study, to evaluate the clinical utility of MSUS in assessing disease activity and classifying disease subtype in JIA, we retrospectively collected patient records, including the values of PGA and CHAQ, laboratory data, and their concomitant MSUS parameters in National Taiwan University Children’s Hospital (NTUCH). The MSUS features in different subtypes of JIA were compared. We then analyzed the correlations between each MSUS parameter, particularly the parameters of a single selected indicator joint, and the results of PGA and CHAQ, and various laboratory data.

## Methods

### Patients

Based on the International League of Associations for Rheumatology (ILAR) diagnostic criteria, children with JIA receiving regular treatment and follow-up at NTUCH from March 2018 to August 2019 were retrospectively recruited into this study. The inclusion criteria included those patients with JIA visited pediatric rheumatic clinics and evaluated by the same pediatric rheumatologist (Dr. Y.H. Yang); The CHAQ assessment was completed by patients themselves and/or their caregivers during the same visit; MSUS examinations and blood tests were then arranged and performed. Of note, above physician’s evaluation, CHAQ assessment, MSUS examination and blood tests were routine practices at NTUCH pediatric rheumatic clinics. MSUS and blood tests were performed at disease onset, once exacerbations were noted, or every 3 to 6 months if stationary. Patients with shoulder, axial skeleton, and hip joint involvement were excluded, because the ultrasound scale we currently used could not access these joints, and the ultrasound probe was incapable of evaluating deeper joints. In addition, considering that extra-articular symptoms and signs were more complicated in systemic JIA and may affect the overall disease activity evaluation, those children with such subtype were excluded in this study. Patients with non-systemic JIA who had uveitis were also excluded. This study has been approved by National Taiwan University’s Hospital Research Ethics Committee (IRB approval number: 202003066RINB).

### Clinical and laboratory assessments

Clinical and laboratory data were collected from patient medical records. The following basic data, including sex, age, and ILAR category, were recorded for each patient. Disease activity evaluation was performed by one pediatric rheumatologist who has worked in this field for more than 20 years. He rated the overall disease activity by PGA according to chief complaints, symptoms, signs, and the findings of physical examinations. The PGA was given as a numerical score on a visual analogue scale (VAS) of 0–100 mm (where 0 = no disease activity and 100 = maximum disease activity). The CHAQ was adapted from the Stanford Health Assessment Questionnaire for assessing functional ability in patients with JIA [18]. The score is called CHAQ disability index (DI), which ranged from 0 to 3. The laboratory tests included white cell count (WBC), platelet count (PLT), hemoglobin (Hb), erythrocyte sedimentation rate (ESR), C-reactive protein (CRP), complement (C)3, and C4. Current status was recorded as active disease or remission according to Wallace criteria [19]. Patients visiting clinics for the first diagnosis/onset, exacerbations, or regular follow-up were further recorded.

### Ultrasound evaluation

**Fig. 1:**
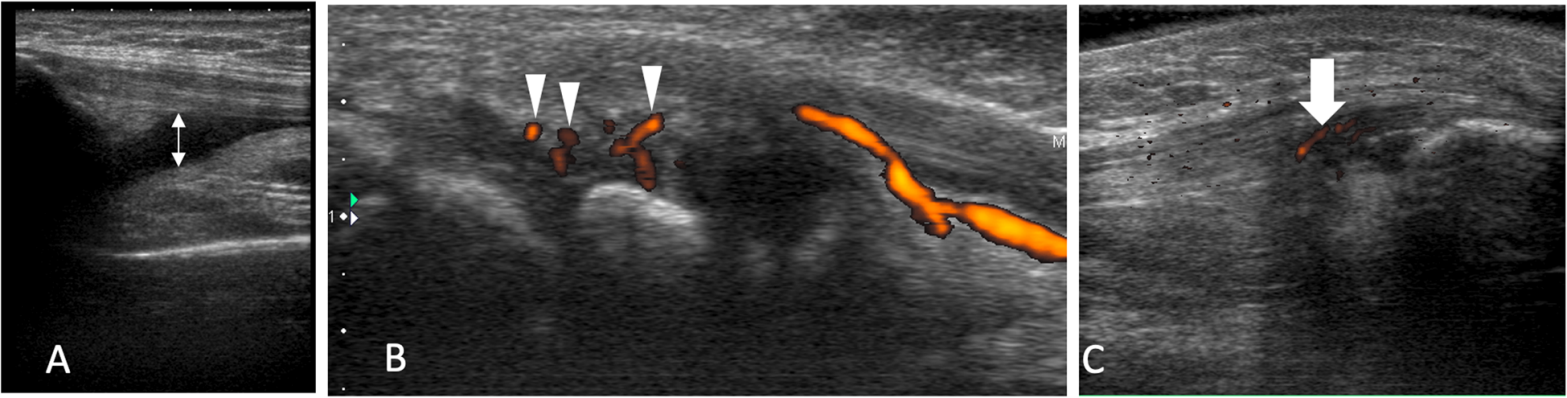
MSUS features in JIA. **a** Effusion (double-headed arrow) in the suprapatellar pouch of the knee. **b** Synovial hypertrophy with PD signals (arrowheads) in the radiocarpal and intercarpal joint. **c** Longitudinal ultrasound image of the patellar tendon that shows hypoechogenicity and PD signals (arrow) inside the enthesis

The pediatric rheumatologist arranged MSUS for the involved joints only, which were determined by the physician according to the patient’s chief complaint and physical examination. The examination was then conducted within 30 min by one rheumatologist (Dr. K.J. Li) who has more than 15 years of experience with MSUS. He has certification in advanced European League Against Rheumatism (EULAR) ultrasound course, including pediatric musculoskeletal ultrasound course, and he is also an advisor of pediatric MSUS in Taiwan. He was blinded to the exact disease status of these JIA patients. Standardized scanning of the joints was based on the recommendations by the Outcomes Measure in Rheumatology (OMERACT) pediatric ultrasound group [20] and EULAR [21]. It took an average of 10 min to evaluate a joint. He then reviewed the images and completed the reports soon after the MSUS examination. The Toshiba Xario XG ultrasound system machine was used with a broadband 7.2–18 MHz linear array transducer and identical settings optimized for power Doppler (PD) for demonstrating superficial structures such as tendons, ligaments, and small joints (standardized presetting, including color gain = 40 and color velocity = 4.7 cm/s; pulse repetition frequency range from 9.8 to 16.5 kHz, that is automatically judged by the machine depending on the joint examined). We recorded the MSUS findings including effusion, synovial hypertrophy, and enthesopathy (Fig. 1) from patients’ MSUS reports that were attached to their medical records. The severity of effusion and synovial hypertrophy was rated by grey-scale (GS) from 0 to 3, and the severity of power signal was rated by PD from 0 to 3. This scoring system was based on the OMERACT pediatric ultrasound task force definition [22]. Subsequently, we calculated the sum of GS and PD as GSPD (GS + PD = GSPD). Among the involved joints of the same subject, the joint with the highest GSPD was selected as the indicator joint. If there were 2 joints with the same GSPD score, we selected the joint with higher PD score as the indicator joint. The GS, PD, and GSPD of this indicator joint were evaluated for their correlations with other parameters and disease status.

### Statistical analysis

Statistical analyses were performed using SPSS statistics version (International Business Machines Corp., Armonk, New York, USA). The age, clinical assessment (PGA score and CHAQ-DI), MSUS parameters (GS, PD, and GSPD), and laboratory data were expressed as mean ± standard deviation (SD). Correlations among clinical assessment and MSUS parameters and laboratory data were calculated by Spearman’s rank correlation. Correlations were considered to be strong, moderate, or weak when absolute values of correlation coefficient (∣rho∣) were > 0.7, 0.4–0.7, or < 0.4, respectively. The scatterplot diagram was used to show the relationship between PGA score and CHAQ-DI. One-way analysis of variance (ANOVA) test was used for comparison of the PGA score among the JIA subtypes. The MSUS features in different subtypes of JIA were examined using the Fisher’s exact test. In these statistical analyses, a *p* value< 0.05 was considered to be statistically significant.

## Results

### Patient and joint characteristics

During the study period, there were 46 non-systemic JIA patients without uveitis receiving regular follow-up at Dr. Yang’s clinics, of which 11 were excluded due to shoulder, axial skeleton, or hip joint involvement. Thirty-five patients were enrolled in this study with a total of 62 visits. Among the 35 patients, 21 were girls and 14 were boys; 13 patients had oligoarthritis (11 patients had persistent oligoarthritis, and 2 had extended oligoarthritis), 15 patients had polyarthritis (4 patients had positive rheumatoid factor (RF) and 11 had negative RF), and 7 patients had enthesitis-related arthritis (ERA). There were 16 patients with one visit, 12 patients with 2 visits, 6 patients with 3 visits, and 1 patient with 4 visits. The mean age on the visiting day was 14.09 years old. The gender ratio of 62 patients on visits was 40:22 (Table 1). Fifteen patients on visits were in remission state (remission on medication), the other 47 patients on visits were in active state. Of patients in active state, 9 were evaluated in the first diagnosis/onset, while the others were followed regularly. As shown in Table 1, 29 patients on visits were treated by non-steroidal anti-inflammatory drugs (NSAIDs), 35 by disease-modifying anti-rheumatic drugs (DMARDs), and 35 by biologics. At each visit, a total of 1 to 12 involved active joints were scanned, which was depended on JIA subtypes and disease status at that time. Finally, a single joint with highest GSPD among all involved active joints was selected as the indicator joint. Therefore, 62 indicator joints were finally recruited for the analysis. Twenty-four joints were derived from JIA patients with oligoarthritis, 29 joints from JIA patients with polyarthritis, and 9 joints from JIA patients with ERA. Among these, 27 were knees, 18 were wrists, 8 joints were elbows, 6 were ankles, 2 were fingers, and 1 was a toe (Table 1).

**Table 1:** Demographic and clinical characteristics of 62 visits

Age (years): mean ± SD	14.09 ± 5.30
Gender: female/male	40/22
JIA subtype: n (%)	
Oligoarthritis	24 (38.7%)
Polyarthritis	29 (46.8%)
ERA	9 (14.5%)
Selected indicator joint: n (%)	
Knee	27 (43.5%)
Wrist	18 (29%)
Elbow	8 (12.9%)
Ankle	6 (9.7%)
Finger	2 (3.2%)
Toe	1 (1.6%)
Treatment: n (%)	
NSAIDs	29 (46.7%)
DMARDs:	35 (56.5%)
Methotrexate	33 (53.2%)
Sulfasalazine	1 (1.6%)
Azathioprine	1 (1.6%)
Biologics:	35 (56.5%)
Etanercept	17 (27.4%)
Adalimumab	16 (25.8%)
Tocilizumab	2 (3.2%)

*JIA* Juvenile idiopathic arthritis, *ERA* Enthesitis-related arthritis, *SD* Standard deviation, *NSAIDs* Non-steroidal anti-inflammatory drugs, *DMARDs* Disease-modifying anti-rheumatic drugs

### Disease activity and physical function scores

**Fig. 2:**
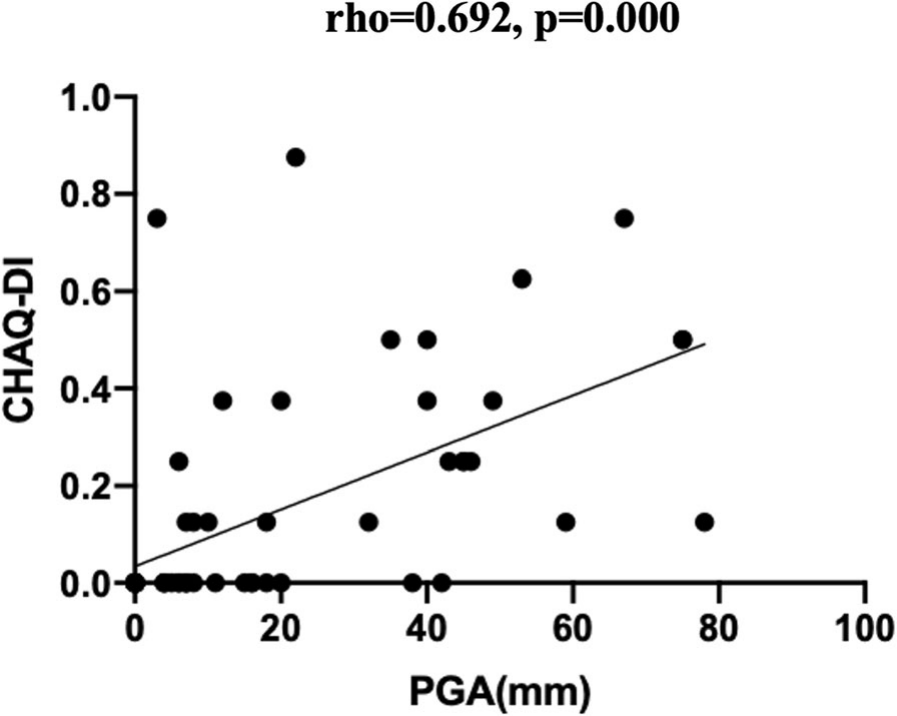
The scatterplot diagram of the correlation between PGA score and CHAQ-DI

JIA disease activity was shown as PGA score, while the physical function was presented as CHAQ-DI. The PGA score and CHAQ-DI of 62 visits were 18.77 ± 22.41 and 0.14 ± 0.88, respectively. The PGA score among the JIA subtypes showed no significant difference (F = 2.043, *p* = 0.139). As can be seen in Fig. 2, the disease activity parameter PGA score had a positive correlation with the physical function parameter CHAQ-DI (rho = 0.692), indicating that the status of disease activity evaluated by a physician was consistent with the reported functional disability in patients with JIA.

### The MSUS features of indicator joint in different subtypes of JIA

**Fig. 3:**
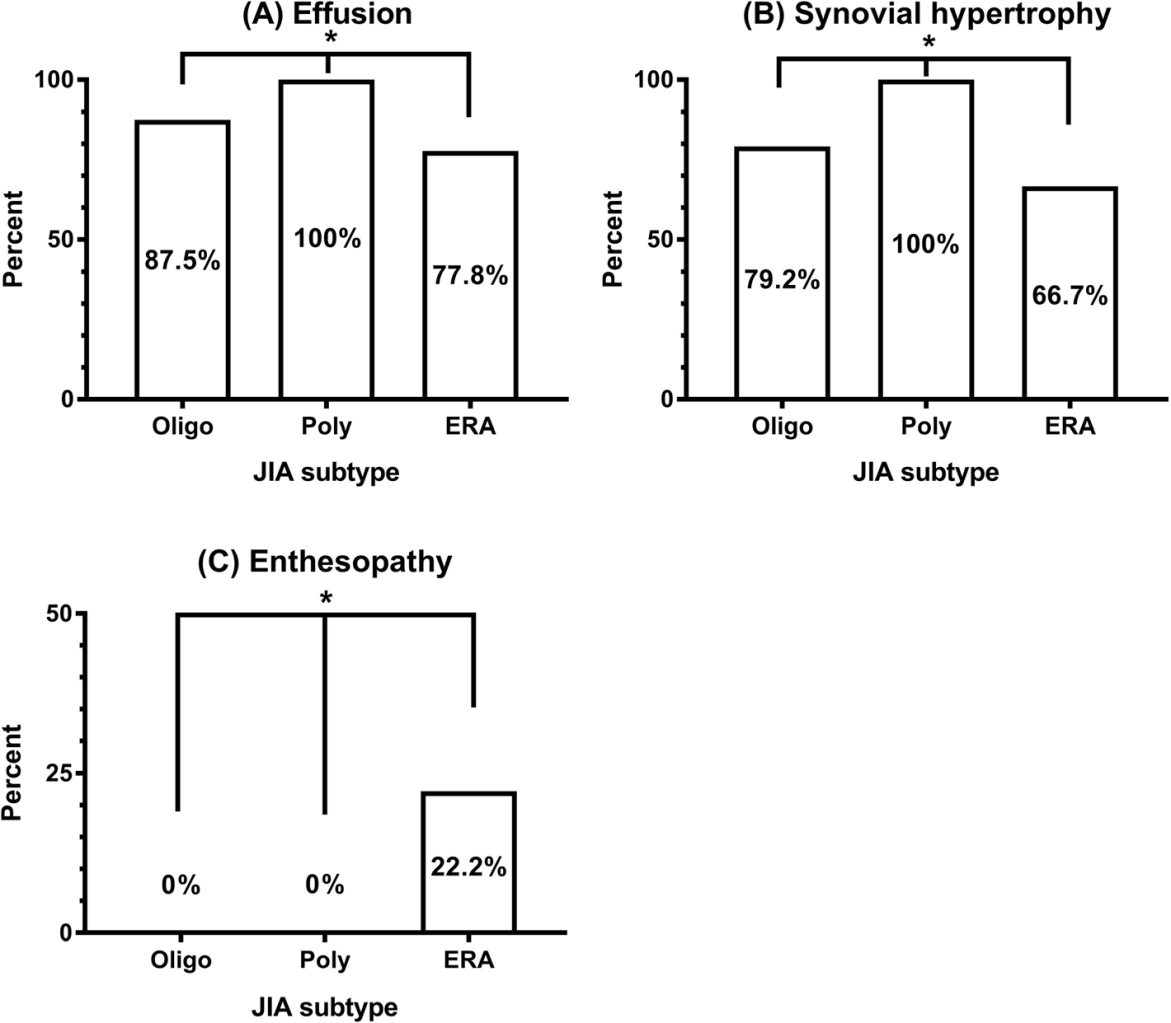
The percentage of (**a**) effusion, (**b**) synovial hypertrophy, and (**c**) enthesopathy in different JIA subtypes. *p < 0.05. Oligo: oligoarthritis, Poly: polyarthritis, ERA: enthesitis-related arthritis

Effusion, synovial hypertrophy, and enthesopathy are main MSUS features of involved joints of JIA [14, 23]. Figure 3 summarized the presence of above 3 features in joints of different subtypes. Of the 62 indicator joints, all joints (29/29) of patients with polyarthritis were characterized by the presence of effusion and synovial hypertrophy. Of the 24 joints from patients with oligoarthritis, 21 (87.5%) and 19 (79.2%) joints were detected with effusion and synovial hypertrophy, respectively. Compared with patients with polyarthritis and patients with oligoarthritis, effusion and synovial hypertrophy were less seen in joints of patients with ERA, 7 of 9 (77.8%) and 6 of 9 (66.7%), respectively. However, enthesopathy was only seen in joints of patients with ERA (2/9) but not in joints of those with polyarthritis (0/29) and oligoarthritis (0/24).

### The correlations between MSUS parameters of indicator joints and laboratory data

The mean values of GS, PD, and GSPD of the 62 indicator joints were 1.74 ± 0.89, 0.53 ± 0.82, and 2.27 ± 1.48. The details of 62 indicator joints were shown in the supplementary table. Since chronic inflammation usually leads to leukocytosis, anemia, thrombocytosis, and elevated ESR, CRP, C3, and C4 [24], laboratory tests including WBC, Hb, PLT, ESR, CRP, C3, and C4 are routinely performed at our clinics to provide another objective parameters for JIA evaluation. The data of the 62 visits showed WBC: 7.93 ± 2.19 × 10^3^/μL, Hb: 12.62 ± 1.67 g/dL, PLT: 340 ± 94 × 10^3^/μL, CRP: 0.53 ± 0.99 mg/dL ESR: 25.22 ± 19.63 mm/hr., C3: 119.3 ± 32.5 mg/dL, C4: 25.4 ± 18.0 mg/dL. We then analyzed the relationship between MSUS parameters (GS/PD/GSPD) and the above laboratory data. As shown in Table 2, GS was weakly correlated with WBC, PLT, C3, and C4. PD had a weak negative correlation with Hb and a weak positive correlation with C4. Moreover, GSPD had a weak positive correlation with WBC and C3, a weak negative correlation with Hb, while had a moderate positive correlation with C4. ESR and CRP, the two common inflammatory parameters, however, were not significantly correlated with GS, PD, or GSPD.

**Table 2:** The correlations between MSUS parameters and laboratory data

	Mean ± SD	GS rho (*p*)	PD rho (*p*)	GSPD rho (*p*)
WBC (10^3^/μL)	7.93 ± 2.19	0.335 (0.013)	0.174 (0.209)	0.315 (0.020)
Hb (g/dL)	12.62 ± 1.67	− 0.200 (0.148)	− 0.282 (0.039)	− 0.280 (0.040)
PLT (10^3^/μL)	340 ± 94	0.300 (0.028)	0.151 (0.277)	0.264 (0.054)
CRP (mg/dL)	0.53 ± 0.99	0.151 (0.275)	0.093 (0.502)	0.154 (0.267)
ESR (mm/hr)	25.22 ± 19.63	0.193 (0.162)	0.211 (0.126)	0.232 (0.091)
C3 (mg/dL)	119.3 ± 32.5	0.322 (0.031)	0.186 (0.221)	0.300 (0.045)
C4 (mg/dL)	25.4 ± 18.0	0.392 (0.008)	0.322 (0.031)	0.428 (0.003)^a^

*GS* Grey-scale, *PD* Power Doppler, *GSPD* The sum of grey-scale and power Doppler, *WBC* White blood cell, *Hb* Hemoglobin, *PLT* Platelet, *CRP* C-reactive protein, *ESR* Erythrocyte sedimentation rate, *C* Complement, *p* = *p* value

^a^ Moderate correlation (0.4 ≤ ∣rho∣ ≤ 0.7)

### The correlations between MSUS/laboratory parameters and JIA disease status

In each visit, there were 10 objective parameters of one patient with JIA including GS, PD, and GSPD of the indicator joint and 7 laboratory data (WBC, Hb, PLT, ESR, CRP, C3, and C4). Their relationship with JIA disease status that included disease activity (PGA score) and physical function (CHAQ-DI) was further elucidated. The results showed PD, WBC, ESR, CRP, and C4 had a weak positive correlation with the PGA score, while GS and GSPD had a moderate positive correlation with the PGA score. On the other hand, GS, PD, GSPD, CRP, and C4 were weakly correlated with CHAQ-DI. The other parameters had no significant correlations with CHAR-DI (Table 3).

**Table 3:** The correlations between MSUS/laboratory parameters and disease status

	PGA rho (*p*)	CHAQ rho (*p*)
GS	0.405 (0.001)^a^	0.257 (0.047)
PD	0.323 (0.010)	0.305 (0.018)
GSPD	0.434 (0.000) ^a^	0.332 (0.010)
WBC (10^3^/μL)	0.28 (0.04)	0.237 (0.084)
Hb (g/dL)	−0.193 (0.161)	− 0.264 (0.054)
PLT (10^3^/μL)	0.045 (0.748)	0.069 (0.622)
CRP (mg/dL)	0.374 (0.005)	0.368 (0.006)
ESR (mm/hr)	0.313 (0.021)	0.215 (0.118)
C3 (mg/dL)	0.283 (0.059)	0.214 (0.158)
C4 (mg/dL)	0.365 (0.014)	0.391 (0.008)

*PGA* Physician Global Assessment, *CHAQ* Childhood Health Assessment Questionnaire, *GS* Grey-scale, *PD* Power Doppler, *GSPD* The sum of grey-scale and power Doppler, *WBC* White blood cell, *Hb* Hemoglobin, *PLT* Platelet, *CRP* C-reactive protein, *ESR* Erythrocyte sedimentation rate, *C* Complement, *p* = *p* value

^a^ Moderate correlation (0.4 ≤ ∣rho∣ ≤ 0.7)

## Discussion

The PGA is a simple and easy tool to assess the disease activity [5], and it can also be used to evaluate the treatment outcome in JIA [25, 26]. However, PGA is a subjective and physician dependent evaluation. Medical ultrasound is currently widely used to create images of internal structures including tendons, muscles, and joints. MSUS is non-invasive that provides images in real-time and does not use harmful ionizing radiation, it is a quick and friendly tool without limitations on language or culture. Therefore, it is accepted not only by children but also their parents. The major disadvantage of MSUS for children is the necessity of a well-trained and qualified operator [14, 27]. In clinical assessment, it can detect subclinical synovitis more frequently than the physical examination [10, 15]. However, the studies about the relationship between MSUS findings and JIA disease activity are few.

Spârchez et al. identified the area with the most pronounced PD activity in 32 patients and they found a high level of agreement between PGA and PD score by Kappa statistics [16]. They didn’t investigate the relationship between GS and PGA in the study. Algergawy et al. selected the knee joints as their objective and detected the synovial thickness and effusion volume in 20 patients with JIA by ultrasound, and they found synovial thickness had a strong correlation with disease activity score of 28 joint count (DAS28) but did not have a correlation with PGA, and effusion volume had a strong correlation with DAS28 and a moderate correlation with PGA [17]. Synovial thickness and effusion volume, like GS, were tools to quantify the severity of synovial hypertrophy and effusion. Their study, however, did not evaluate PD activity and may be useful only in JIA patients with knee involvement. In our study, we simultaneously analyzed GS, PD, and GSPD for their correlations with disease activity, and found GS and GSPD of the indicator joints had a moderate correlation with the PGA score.

In contrast to our results, Magni-Manzoni et al. showed poor correlations between MSUS parameters and PGA, and even the Juvenile Arthritis Disease Activity Score of 52 joint count (JADAS52) in 32 patients with JIA [15]. In that study, they used the sum of MSUS parameters of 52 joints of each patient to compare the clinical parameters, but we used the MSUS parameters of the indicator joint to analyze. In fact, JIA is a disorder comprising a clinically heterogeneous group of chronic arthritis with different subtypes and affected joint counts [28]. One study showed the initial average active joint counts in persistent oligoarthritis and in RF negative polyarthritis were 1 and 8 [29]. In our clinical experience, although the affected joint count in oligoarthritis was fewer than that in polyarthritis, the disease severity in oligoarthritis may not be less than that in polyarthritis. Therefore, the usage of the sum of MSUS parameters of the involved joints may underestimate the disease severity in the subtypes with less affected joints. To avoid this possibility, we used one single indicator joint in this study rather than all active joints for subsequent analysis.

Power Doppler signal in synovial tissue reflexes hypervascularization of the synovial tissue, which is considered as an active state [30]. Magni-Manzoni et al. found the patients with JIA with persistent inactive disease had a greater frequency of PD signal at the beginning of the study than the patients with synovitis flare, which suggested PD signal did not predict subsequent synovitis flare [31]. Recently, Miotto e Silva et al. found the risk of flare was five times higher in patients with JIA with positive PD signal in clinical remission than in patients without positive PD signal [32]. The uncertain role of PD signal in JIA may be due to the different sensitivity of PD signal in younger children and adolescent and due to the difference in immunopathological mechanism between JIA and seropositive RA [32, 33]. In our study, the combination of PD score and GS score (GSPD) seemed to have a better correlation than a single MSUS parameter (GS or PD) with disease activity (Table 3), which suggested that the evaluation of PD in JIA was still important and may have a synergistic effect with GS on disease evaluation. To our knowledge, our study is the first to use the highest GSPD score of the involved joints to assess disease activity in JIA.

Previously, some laboratory parameters, particularly ESR and CRP have been used to assist the evaluation of JIA disease activity [34, 35]. In our study, however, there were no any strong or moderate correlations between laboratory parameters and disease activity. Similar results were noted in the study of Berntson et al. [6]. Among all parameters, although some correlations existed between laboratory and MSUS parameters, GSPD presented a best correlation with the PGA score (with highest rho value). It indicated that MSUS may be a better tool to detect disease activity than laboratory tests.

In addition to disease activity evaluated by PGA, daily physical functions of patients with JIA were assessed in our study by patients themselves and/or caregivers. Several studies showed the CHAQ was useful for assessing functional ability rather than disease activity [25, 36, 37]. Of note, a moderate correlation was found between CHAQ-DI and PGA score (Fig. 2). Together, although evaluated by different perspectives, tools, and investigators, the overall JIA disease status was likely to be consistent in this study. Thus, we also evaluated the relationship between each parameter and physical function, and found that, although not strongly associated, 3 MSUS parameters of the indicator joints, GS, PD, and GSPD, and 2 laboratory parameters, CRP and C4, had weak positive correlations with CHAQ-DI.

The MSUS features in different JIA subtypes were analyzed. Effusion and synovial hypertrophy were seen most in polyarthritis, while least seen in ERA. Enthesopathy was only seen in ERA, although the case number was small (Fig. 3). MSUS may help in JIA classification. Previous studies also showed the importance of MSUS in ankles to differentiate between synovitis and tenosynovitis and to improve classification in JIA [38, 39]. Jousse-Joulin et al. reported 9.4% (20/213) of entheseal sites had enthesitis in patients with JIA [40], and Weiss et al. reported 57% (17/30) of the patients with ERA had enthesopathy on MSUS examination [12]. The reason for only having 22.2% (2/9) of ERA joints with enthesopathy could be that the study included the findings of indicator joints, but not all involved joints.

There are some limitations in our study. Only 3 subtypes of JIA, oligoarthritis, polyarthritis, and ERA were recruited. We excluded the patients with shoulder, axial skeleton, and hip joint involvement. As a result, the case number was limited. The mean age of total patients on visits was 14.09 ± 5.30 years, only 9 of the 62 patients on visits were less than 7 years old. The findings of this study may not be generalized for younger children. Recruitment of more JIA cases including younger patients is necessary to validate the current results. In this study, performing MSUS to only involved joints as was suggested by the pediatric rheumatologist that may be considered as a bias. Furthermore, PGA is a simple tool for JIA activity evaluation and is easily applied in daily practice, however, it is just one of a core set of JADAS, which has been widely used in many studies. The correlations between MSUS parameters of the indicator joints and JADAS should be investigated in the future.

## Conclusions

Although more cases and further studies are needed, the current study revealed that MSUS parameters of a single indicator joint in non-systemic JIA showed significant correlations with PGA. MSUS is an acceptable non-invasive tool for patients with JIA that might assist disease classification, and whose parameters of indicator joints may potentially contribute to the disease activity evaluation.

## Supplementary Information

**Additional file 1: Supplementary table.** MSUS parameters in 62 indicator joints.

## Data Availability

The data are available on request to the corresponding author.

## Abbreviations

JIA: Juvenile idiopathic arthritis
CHAQ: Childhood Health Assessment Questionnaire
PGA: Physician Global Assessment
MSUS: Musculoskeletal ultrasound
RA: Rheumatoid arthritis
VAS: Visual analogue scale
DI: Disability index
WBC: White cell count
PLT: Platelet count
Hb: Hemoglobin
ESR: Erythrocyte sedimentation rate
CRP: C-reactive protein
C: Complement
GS: Grey-scale
PD: Power Doppler
RF: Rheumatoid factor
ERA: Enthesitis-related arthritis
JADAS: Juvenile Arthritis Disease Activity Score
EULAR: European League Against Rheumatism
OMERACT: Outcomes Measure in Rheumatology
NSAIDs: Non-steroidal anti-inflammatory drugs
DMARDs: Disease-modifying anti-rheumatic drugs
