# Supplementary material for: Utilizing ultrasound findings of a single indicator joint to assess non-systemic juvenile idiopathic arthritis
Source: Pediatr Rheumatol Online J. 2021 Apr 29;19:60. doi: 10.1186/s12969-021-00550-0 (PMC8082904; doi:10.1186/s12969-021-00550-0)
Supplement: Supplementary file 1 — Additional file 1: Supplementary table. MSUS parameters in 62 indicator joints. [file 12969_2021_550_MOESM1_ESM.docx]

**Supplementary table. MSUS parameters in 62 indicator joints**

|  |  | GS | PD | GSPD |
| --- | --- | --- | --- | --- |
|  | 1 | 3 | 3 | 6 |
|  | 2 | 3 | 0 | 3 |
|  | 3 | 1 | 0 | 1 |
|  | 4 | 3 | 0 | 3 |
|  | 5 | 2 | 0 | 2 |
|  | 6 | 0 | 0 | 0 |
|  | 7 | 3 | 2 | 5 |
|  | 8 | 1 | 0 | 1 |
|  | 9 | 2 | 2 | 4 |
|  | 10 | 2 | 0 | 2 |
|  | 11 | 1 | 1 | 2 |
| Oligoarthritis | 12 | 0 | 0 | 0 |
|  | 13 | 1 | 0 | 1 |
|  | 14 | 3 | 2 | 5 |
|  | 15 | 1 | 0 | 1 |
|  | 16 | 1 | 0 | 1 |
|  | 17 | 2 | 0 | 2 |
|  | 18 | 2 | 0 | 2 |
|  | 19 | 0 | 0 | 0 |
|  | 20 | 2 | 0 | 2 |
|  | 21 | 2 | 1 | 3 |
|  | 22 | 1 | 0 | 1 |
|  | 23 | 1 | 0 | 1 |
|  | 24 | 2 | 0 | 2 |
|  | 1 | 2 | 0 | 2 |
|  | 2 | 2 | 1 | 3 |
|  | 3 | 3 | 1 | 4 |
|  | 4 | 3 | 1 | 4 |
|  | 5 | 3 | 1 | 4 |
|  | 6 | 2 | 0 | 2 |
|  | 7 | 1 | 0 | 1 |
|  | 8 | 3 | 2 | 5 |
|  | 9 | 2 | 1 | 3 |
|  | 10 | 2 | 0 | 2 |
|  | 11 | 2 | 0 | 2 |
|  | 12 | 2 | 1 | 3 |
|  | 13 | 1 | 0 | 1 |
| Polyarthritis | 14 | 2 | 3 | 5 |
|  | 15 | 2 | 1 | 3 |
|  | 16 | 3 | 0 | 3 |
|  | 17 | 1 | 0 | 1 |
|  | 18 | 2 | 2 | 4 |
|  | 19 | 3 | 2 | 5 |
|  | 20 | 1 | 0 | 1 |
|  | 21 | 2 | 0 | 2 |
|  | 22 | 2 | 0 | 2 |
|  | 23 | 1 | 0 | 1 |
|  | 24 | 1 | 0 | 1 |
|  | 25 | 3 | 1 | 4 |
|  | 26 | 2 | 1 | 3 |
|  | 27 | 3 | 0 | 3 |
|  | 28 | 1 | 0 | 1 |
|  | 29 | 2 | 1 | 3 |
|  | 1 | 2 | 2 | 4 |
|  | 2 | 0 | 0 | 0 |
|  | 3 | 2 | 1 | 3 |
|  | 4 | 2 | 0 | 2 |
| ERA | 5 | 0 | 0 | 0 |
|  | 6 | 1 | 0 | 1 |
|  | 7 | 1 | 0 | 1 |
|  | 8 | 1 | 0 | 1 |
|  | 9 | 1 | 0 | 1 |
| mean ± SD |  | 1.74±0.89 | 0.53±0.82 | 2.27±1.48 |

GS: grey-scale, PD: power Doppler, GSPD: the sum of grey-scale and power Doppler, ERA: enthesitis-related arthritis, SD: standard deviation
